# High-efficiency coherence-preserving harmonic rejection with crystal optics

**DOI:** 10.1107/S1600577518009645

**Published:** 2018-08-14

**Authors:** Fan Zhang, Andrew J. Allen, Lyle E. Levine, Gabrielle G. Long, Ivan Kuzmenko, Jan Ilavsky

**Affiliations:** a Material Measurement Laboratory, National Institute of Standards and Technology, 100 Bureau Drive, Gaithersburg, MA 20899, USA; b X-ray Science Division, Advanced Photon Source, Argonne National Laboratory, Argonne, IL 60439, USA

**Keywords:** harmonic rejection, monochromator, coherent X-ray scattering, SAXS, XAFS

## Abstract

This work reports a harmonic-rejection scheme based on the combination of Si(111) monochromator and Si(220) harmonic-rejection crystal optics. This approach is of importance to a wide range of X-ray applications in all three major branches of modern X-ray science (scattering, spectroscopy, imaging) based at major facilities, and especially relevant to the capabilities offered by the new diffraction-limited storage rings. It was demonstrated both theoretically and experimentally that, when used with a synchrotron undulator source over a broad range of X-ray energies of interest, the harmonic-rejection crystals transmit the incident harmonic X-rays on the order of 10^−6^. Considering the flux ratio of fundamental and harmonic X-rays in the incident beam, this scheme achieves a total flux ratio of harmonic radiation to fundamental radiation on the order of 10^−10^. The spatial coherence of the undulator beam is preserved in the transmitted fundamental radiation while the harmonic radiation is suppressed, making this scheme suitable not only for current third-generation synchrotron sources but also for the new diffraction-limited storage rings where coherence preservation is an even higher priority. Compared with conventional harmonic-rejection mirrors, where coherence is poorly preserved and harmonic rejection is less effective, this scheme has the added advantage of lower cost and footprint. This approach has been successfully utilized at the ultra-small-angle X-ray scattering instrument at the Advanced Photon Source for scattering, imaging and coherent X-ray photon correlation spectroscopy experiments. With minor modification, the harmonic rejection can be improved by a further five orders of magnitude, enabling even more performance capabilities.

## Abstract 2

A coherence-preserving harmonic-rejection scheme based on parallel crystal optics that achieves a total flux ratio of harmonic radiation to fundamental radiation on the order of 10^−10^ or lower is reported.

## 1. Introduction   

Synchrotron X-ray sources and the experimental capabilities that they enable are increasingly important in many scientific fields, including physics, materials science, chemistry, environmental science and biology. For most synchrotron-based experimental techniques, monochromatization of the X-ray radiation is required. Crystal-based monochromators are essential components of modern X-ray beamline design which, when properly configured in accordance with source characteristics, can deliver high-brilliance X-rays of specific wavelength (energy) to the sample position. The monochromatization process follows Bragg’s law, *nλ* = 2*d*sin(θ_B_), where *n* is a positive integer, *λ* is the X-ray wavelength, *d* is the lattice spacing of the monochromator crystals and θ_B_ is one half of the diffraction angle 2θ_B_. Following Bragg’s law, it is obvious that the monochromator allows through not only X-rays of fundamental radiation with wavelength *λ* (where *n* = 1) but also harmonic radiation with wavelength *λ*/*n* (excluding crystallographically forbidden reflections) resulting from higher orders of diffraction. For many applications the intensity of the harmonic radiation relative to the fundamental radiation can be significant, and this can lead to distortions and errors in the experimental data. Rejection of the harmonic radiation is desirable and in many cases necessary in techniques across modern X-ray science.(Gauthier *et al.*, 1999 ▸; Paterson *et al.*, 2011 ▸).

A variety of methods have been developed to suppress harmonic radiation, including crystal detuning of a double-crystal monochromator (Bonse *et al.*, 1976 ▸; Hou, 2005 ▸), total-reflection mirrors (Latimer *et al.*, 1995 ▸; Hastings *et al.*, 1978 ▸), asymmetric bent-Laue crystals (Karanfil *et al.*, 2004 ▸), compound refractive lenses (Polikarpov *et al.*, 2014*a* ▸,*b* ▸) and undulator segmentation (Tanaka & Kitamura, 2002 ▸). Among these, crystal detuning and harmonic-rejection X-ray mirrors are the most common. Crystal detuning relies on the fact that the X-ray refractive index of a given material is energy dependent while the bandwidth of the fundamental radiation can be significantly larger than those of the harmonic radiation. Therefore, by detuning the second monochromator crystal slightly, the flux of harmonic radiation can be suppressed by 10^−2^ to 10^−3^ of their original values (Hou, 2005 ▸). This level of harmonic rejection, though good enough sometimes, is often inadequate, leading to the need for other harmonic-rejection devices, most commonly total-reflection X-ray mirrors in tandem with crystal detuning to further suppress harmonic radiation. These X-ray mirrors are often coated with a high-atomic-number (high-*Z*) material, and can be oriented at a selected incident angle so that incident X-rays below a specific energy are totally reflected while harmonic radiation is absorbed. Such mirrors generally have multiple stripes of different reflecting materials to enable harmonic rejection over a wide energy range. Their harmonic-rejection efficiency, defined as the flux ratio of harmonic radiation to fundamental radiation, can reach the order of 10^−3^ (Bilderback & Hubbard, 1982 ▸). Combining crystal detuning with total-reflection X-ray mirrors, a harmonic-rejection efficiency ranging from 10^−5^ to 10^−6^ can be achieved (Lingham *et al.*, 1996 ▸).

With the increasing brightness and coherence of new X-ray sources such as diffraction-limited storage rings and X-ray free-electron lasers, X-ray optics that can define, optimize and preserve beam characteristics, particularly beam coherence, are desirable. Many scientific opportunities enabled by these new sources are highly sensitive to the wavefront distortions caused by X-ray optics. For example, it is estimated that, for the purpose of coherence preservation of hard X-rays, X-ray mirrors need to have a surface finish with slope errors less than 50 nrad and root-mean-square height errors less than 0.5 nm over mirror dimensions approaching 1 m in length along the beam-propagation direction (Pardini *et al.*, 2015 ▸; Mills & Padmore, 2013 ▸). These stringent requirements and high costs associated with constructing mirrors with such low surface figure errors and roughness present major challenges.

In this paper, we offer a highly effective mirrorless harmonic-rejection scheme that has been successfully utilized at the Advanced Photon Source (APS), Argonne National Laboratory, Argonne, IL, USA. This scheme exploits a crystal mismatch between the monochromator crystals and Bonse–Hart-type harmonic-rejection crystals. We demonstrate that this scheme can suppress the intensity of harmonic radiation to ∼10^−10^ of the intensity of the fundamental radiation over a wide energy range, while preserving the spatial coherence of the X-ray beam.

## 2. Theoretical considerations   

The dynamical diffraction of X-rays by a perfect single crystal is well understood. In this paper, we follow the treatment by Als-Nielsen & McMorrow (2011 ▸). The amplitude, *r*, of the single-crystal reflectivity curve, as a function of the real part of *x*_c_, Re(*x*_c_), follows
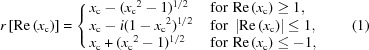
where 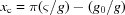, 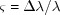 is the relative bandwidth in wavelength *λ*,
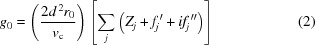
and 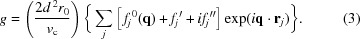
Here *v*_c_ is the volume of the unit cell, *r*_0_ is the scattering amplitude per electron, *d* is the lattice spacing, *f_j_′* and *f_j_*′′are the real and imaginary parts of the dispersion correction to the atomic scattering length *f_j_* ^0^(**q**). Here, *q* = 4π/*λ*sin(*θ*), where *θ* is one half of the scattering angle 2*θ*. We calculated the energy-dependent *f_j_′* using the scattering contrast factor calculator in the small-angle scattering analysis package *Irena* (Ilavsky & Jemian, 2009 ▸; Cromer & Liberman, 1981 ▸). The crystal Darwin curve (*i.e.* the intensity reflectivity curve), expressed as a function of (*θ* – *θ*_B_), is derived from the square of the amplitude given in equation (1). Based on this notation, the angular full width at half-maximum (FWHM) of the Darwin curve follows 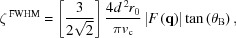
where 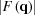 is the unit-cell structure factor in the diffraction direction and is energy dependent. An important feature of the Darwin curve is that its center displays a small angular offset from the Bragg angle. Mathematically, this offset is equal to 
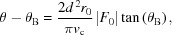
where *F*_0_ is the unit-cell structure factor in the forward direction. For a given Bragg angle, the higher the X-ray energy, the smaller the angular offset and the smaller the FWHM of the Darwin curve. Thus, it is important to note that even nondispersive crystal optics exhibit a small energy dispersion for different crystal reflections.

**Figure 1:**
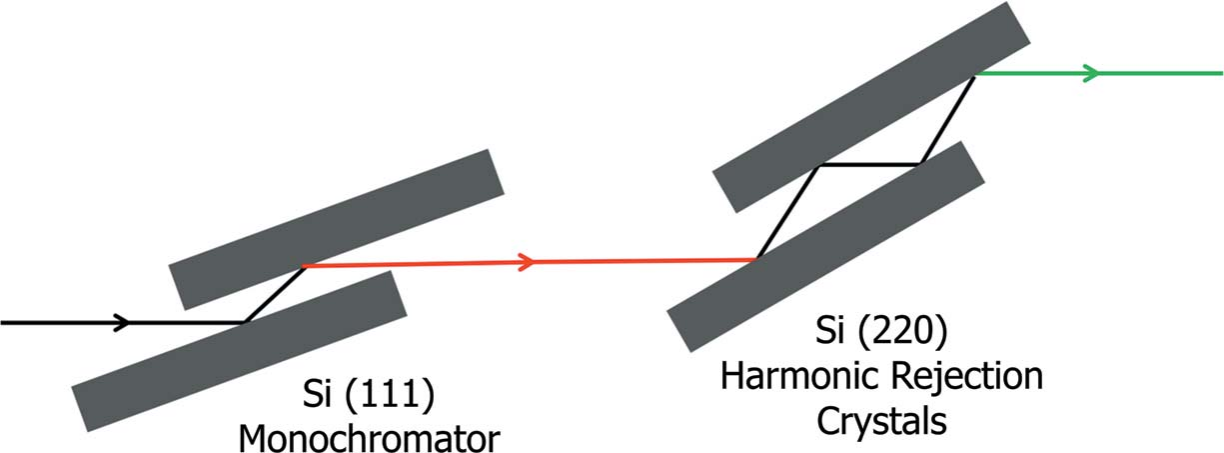
Illustration of the harmonic-rejection scheme reported in this paper. X-rays are reflected twice by the Si(111) monochromator crystals and four times by the Si(220) harmonic-rejection crystals. The two crystals in the Si(111) monochromator and the two Si(220) crystals of the harmonic-rejection pair are perfectly parallel to each other.

Our scheme exploits this offset by regarding the Darwin curve as an angular filter (Zhang *et al.*, 2008 ▸). This scheme is illustrated in Fig. 1 ▸, where X-rays are diffracted twice off the Si(111) monochromator crystals and four times by the Si(220) harmonic-rejection crystals. The mismatch between the monochromator crystal diffraction (111) and the harmonic-rejection crystal diffraction (220) is critical to this scheme. The Si(111) crystal passes spectral contamination following third, fourth, fifth… order diffractions (*n* = 3, 4, 5…). For an undulator gap optimized for fundamental radiation, the X-ray flux of the harmonic radiation drops precipitously as the X-ray energy increases. Notably, second-order diffraction is forbidden for an Si(111) monochromator. Thus, we consider only fundamental radiation with the wavelength *λ* and harmonic radiation with *λ*/3, and we neglect further higher-order harmonic radiation as a result of their much lower flux. As we shall explain in detail later, Si(111) and Si(220) crystal diffractions possess different relative angular offsets between the diffracted fundamental radiation and diffracted harmonic radiation. This point forms the basis of the highly effective harmonic-rejection scheme described in this paper.

**Figure 2:**
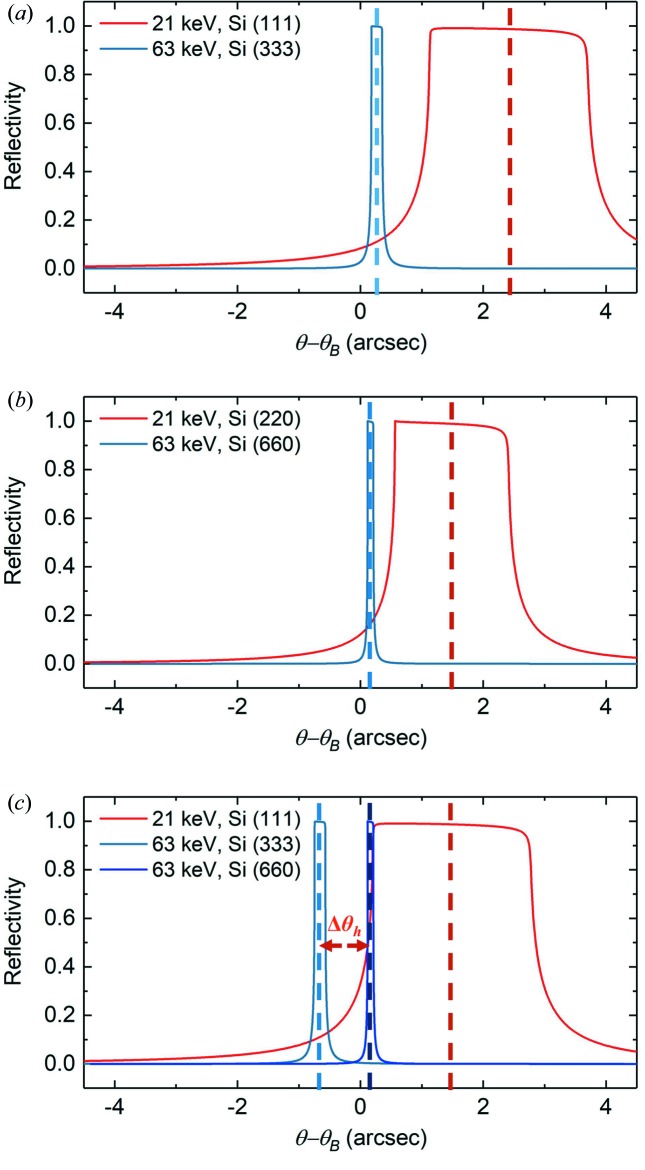
Calculated crystal Darwin curve as a function of angular offset from the Bragg angle for (*a*) 21 keV X-rays from Si(111) crystal planes and 63 keV X-rays from Si(333) crystal planes; (*b*) 21 keV X-rays from Si(220) crystal planes and 63 keV X-rays from Si(660) crystal planes. The red dashed lines in (*a*) and (*b*) show the center of the Darwin curves for 21 keV X-rays with Si(111) and Si(220) crystal planes, respectively. The blue dashed lines in (*a*) and (*b*) show the center of the Darwin curves for 63 keV X-rays with Si(333) and Si(660) crystal planes, respectively. Panel (*c*) demonstrates that, while maximizing the transmission of the fundamental radiation exiting from the Si(111) monochromator, the incident angle of the harmonic radiation (*n* = 3) situates at the tail (Δθ_h_ from the center) of the Si(660) crystal Darwin curve, which leads to the highly effective harmonic-rejection scheme reported in this paper.

We first inspect the general characteristics of the crystal Darwin curves. An example is shown in Fig. 2 ▸(*a*), which compares the calculated crystal Darwin curves as a function of angular offset from the Bragg angles for fundamental radiation of 21 keV X-rays and harmonic radiation of 63 keV X-rays (third-order diffraction). The angular offset 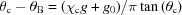. Here, 21 keV X-rays are diffracted by Si(111) planes and 63 keV X-rays are diffracted by Si(333) planes with the same Bragg angle θ_B_. The parameters used to calculate these Darwin curves are listed in Table 1 ▸. It is apparent that the Darwin curve is narrower and its center deviates from θ_B_ by a smaller amount for harmonic radiation than it does for fundamental radiation. The same statements can be made for Fig. 2 ▸(*b*), which shows the calculated crystal Darwin curves as a function of the angular offset from the Bragg angle for fundamental radiation of 21 keV X-rays and harmonic radiation of 63 keV X-rays diffracted by the Si(220) and Si(660) planes, respectively.

The angular offsets, θ_C_−θ_B_, of the 21 keV and 63 keV X-rays from their respective Bragg angles for Si(111) and Si(220) optics are different. The dashed lines in Fig. 2 ▸ are at the centers of the Darwin curves. The Darwin curves for 21 keV X-rays diffracted by Si(111) and 63 keV X-rays diffracted by Si(333) are centered at offsets of 2.4079 arcsec and 0.2671 arcsec, respectively, from the θ_B_ values for the Si(111)/Si(333) diffraction planes. This leads to a relative angular offset of 2.1408 arcsec. Meanwhile, the Darwin curves for 21 keV X-rays diffracted by Si(220) and 63 keV X-rays diffracted by Si(660) are centered at 1.4824 arcsec and 0.1647 arcsec, respectively, from the θ_B_ values for Si(220)/Si(660) diffraction planes, leading to a relative-angular offset of 1.3176 arcsec. This allowed a relative offset between 21 keV and 63 keV X-rays arriving at the harmonic-rejection crystal pair that clearly does not match the relative offset allowed and transmitted for these same X-ray energies by the monochromator.

The X-rays generated by an APS undulator A source are collimated with an angular spread. The angular divergence has a Gaussian width on the scale of 15 µrad (3.1 arcsec) (Dejus *et al.*, 1994 ▸). The flux of the X-ray beam is optimized for fundamental radiation. In our scheme (Fig. 1 ▸) the undulator beam is first received by the Si(111) monochromator. The monochromator is set to monochromatize the fundamental radiation, *i.e.* the incident angle is close to the Bragg angle θ_B_, as dictated by Bragg’s law. Other than the harmonic radiation, X-rays of almost all other energies are not transmitted through the monochromator. Darwin curves also serve as angular filters. As Fig. 2 ▸(*a*) clearly shows, the outgoing directions of the fundamental radiation and harmonic radiation are slightly different. In the case of 21 keV, this angular difference is 2.1408 arcsec.

After exiting the Si(111) monochromator, the X-rays, which now consist of fundamental radiation with wavelength *λ* and harmonic radiation with wavelength *λ*/3 (neglecting harmonic radiation resulting from higher-order diffraction), enter the Si(220) harmonic-rejection crystals. In our scheme, we seek to maximize the intensity of the fundamental radiation after the X-rays exit the Si(220) crystals. To achieve this, the incident angle of the fundamental radiation entering the Si(220) optics must be centered on the center of the Si(220) Darwin curve for the same energy. This is demonstrated by the red dashed lines in Figs. 2 ▸(*b*) and 2(*c*). The Si(111) acceptance angle is larger than that of Si(220), so the Si(220) Darwin curve is fully populated; this offsets the incident angle of the harmonic radiation (*λ*/3) away from the center of the corresponding Si(660) Darwin curve where it could be transmitted through the harmonic-rejection crystal pair. Again, using 21 keV and 63 keV X-rays as an example, the angular difference between the blue dashed lines in Figs. 2 ▸(*b*) and 2(*c*) is 0.8231 arcsec, approximately 8.5 times the FWHM of the Si(660) Darwin curve at 63 keV (0.0973 arcsec).

Following this scheme mathematically, the transmission of the harmonic radiation through the harmonic-rejection crystals is defined as
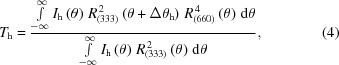
where *I*_h_(θ) is the flux of the incident beam at the monochromator and *R*_(333)_(*θ*) and *R*_(660)_(*θ*) are the Si(333) and Si(660) Darwin curves for harmonic radiation. Δ*θ* = *θ* ^c^_(111)_ − *θ* ^c^_(220)_, where *θ* ^c^_(111)_ and *θ* ^c^_(220)_ are the center of the Si(111) and Si(220) Darwin curves of the fundamental radiation, exampled by the red dashed lines in Figs. 2 ▸(*a*) and 2(*b*), respectively. Consequently, Δθ_h_ = *θ* ^c^_(111)_ − *θ* ^c^_(220)_ − *θ* ^c^_(333)_ + *θ* ^c^_(660)_. Here, *θ* ^c^_(333)_ and *θ* ^c^_(660)_ are the centers of the Si(333) and Si(660) Darwin curves for fundamental radiation, as shown by the blue dashed lines in Figs. 2 ▸(*a*) and 2(*b*). Similarly, the transmission of the fundamental radiation from the harmonic-rejection crystals can be calculated by replacing *R*_(333)_(*θ*) and *R*_(660)_(*θ*) with *R*_(111)_(*θ*) and *R*_(220)_(*θ*) in equation (4), respectively.

One key characteristic of parallel crystal optics is the power-law reduction of the diffracted intensity in the tails of the reflectivity curves, resulting from multiple diffractions by parallel perfect-crystal optics (Bonse & Hart, 1965 ▸). Our scheme takes advantage of this characteristic, where the incident angle of the harmonic radiation at the harmonic-rejection crystal pair occurs at the tail of the corresponding Si(660) Darwin curve that represents the acceptance window for diffraction, as illustrated in Figs. 2 ▸(*b*) and 2(*c*). In our calculation, we further assume that the beam incident at the monochromator is uniform in its angular intensity distribution, *i.e.* *I*_h_(θ) is independent of *θ*. Thus, *I*_h_(θ) in equation (4) cancels out, and the transmission of the third-harmonic X-rays can be calculated using the theoretical Darwin curves alone.

**Figure 3:**
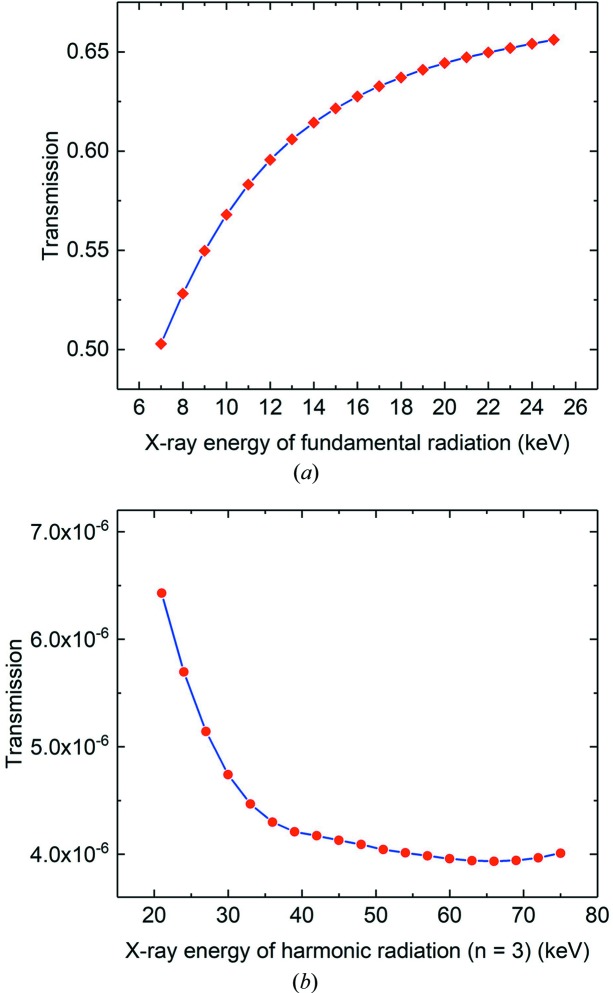
Calculated energy-dependent transmission of (*a*) the fundamental radiation and (*b*) the harmonic radiation (*n* = 3) using the scheme shown in Fig. 1 ▸. This transmission is defined as the flux ratio of the outgoing to the incoming incident harmonic radiation for the harmonic-rejection crystal pair.

Fig. 3 ▸ shows the calculated transmission of the fundamental and harmonic radiations using the harmonic-rejection crystal pair in a fundamental-radiation energy range from 7 keV to 25 keV. The transmission of the fundamental radiation shows a monotonic increase (improved throughput) with increasing X-ray energy. In general, within this energy range, >50% of the desired fundamental radiation is allowed through the harmonic-rejection crystal pair. The transmission of the harmonic radiation is on the order of 10^−6^, and becomes smaller with increasing X-ray energy. The sharp contrast between the transmissions of the fundamental and harmonic radiations clearly demonstrates that the harmonic radiation is preferentially and effectively suppressed. This is assisted by the fact that the incident flux of the harmonic radiation at the monochromator is already a few orders of magnitude lower than that of the fundamental radiation, owing to the undulator radiation characteristics. This fact, combined with the 10^−6^ transmission of the harmonic radiation demonstrated here, leads to an even smaller flux ratio between the harmonic radiation and fundamental radiation after the harmonic-rejection crystal pair. This will be discussed further in the experimental section.

It is worth noting that the forbidden Si(222) reflection from the monochromator does have a very small intensity as a result of the asymmetric distribution of the electronic structure, and this was not explicitly included in the above analysis. However, the small intensity of this forbidden reflection (∼10^−3^), coupled with the harmonic-rejection scheme described above, results in a transmitted intensity that is significantly <10^−6^.

**Figure 4:**
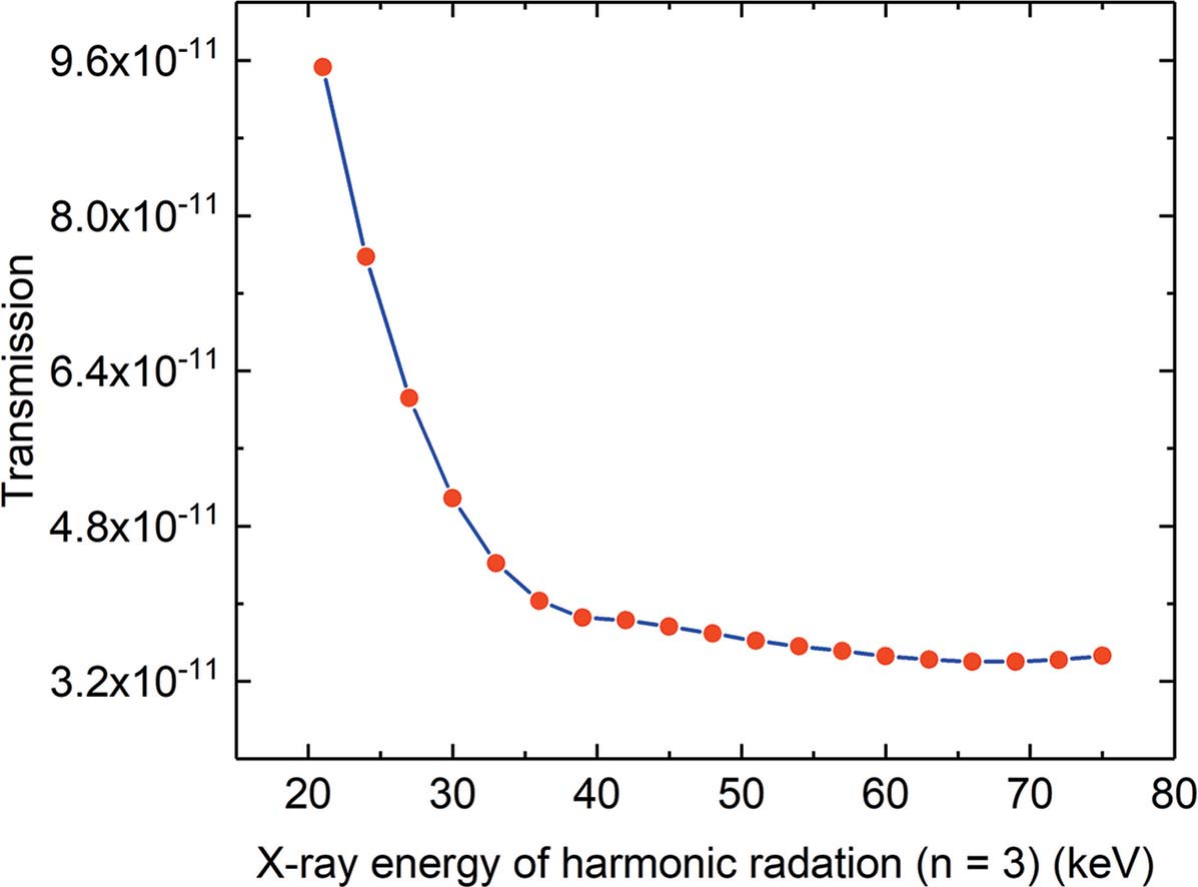
Calculated energy-dependent transmission of the (*n* = 3) harmonic X-rays using a modified scheme with four diffractions by the Si(111) monochromator crystals, as well as four diffractions by the Si(220) harmonic-rejection crystal pair. The harmonic transmission is defined as the flux ratio of the outgoing to the incoming harmonic radiation incident at the harmonic-rejection crystal pair. Compared with Fig. 3 ▸(*b*), it is apparent that the transmission of the (*n* = 3) harmonic X-rays is reduced by another five orders of magnitude by introducing two additional reflections in the Si(111) monochromator.

Although the harmonic rejection is already effective using the scheme shown in Fig. 1 ▸, if more harmonic rejection is required, for example, to exploit opportunities for new instrument designs, the scheme in Fig. 1 ▸ can be adjusted to achieve this. Instead of having two diffractions by the Si(111) monochromator crystals, we can use four. Consequently, the power of *R*_(333)_(*θ*) in equation (4) changes from two to four. The transmission of the fundamental radiation is nearly unchanged as this transmission is largely determined by the relative width of the Darwin curves for Si(111) and Si(220) at the energy corresponding to the fundamental radiation. The transmission of the harmonic radiation, however, decreases by another five orders of magnitude to 10^−11^, as shown in Fig. 4 ▸.

We point out that the key for our scheme to work is the dispersion between the Si(111) optics in the monochromator and the Si(220) optics in the harmonic-rejection crystal pair. Should both crystals be of the same type, for example Si(111), Δθ in equation (4) becomes 0, and both the fundamental and harmonic radiation that exit from the monochromator will be accepted by the angular filters imposed by the harmonic-rejection crystal pair. Thus, effective harmonic rejection will not be achieved under these conditions.

## 3. Experimental verification: harmonic transmission   

We measured the transmission of the fundamental and harmonic radiations for our scheme using the ultra-small-angle X-ray scattering (USAXS) instrument at APS sector 9-ID (Ilavsky *et al.*, 2009 ▸, 2018 ▸). This beamline makes use of an APS undulator A source and a Si(111) double-crystal monochromator. Importantly, the USAXS instrument is equipped with nondispersive Bonse–Hart-type Si(220) crystal optics to collimate the incident X-rays. Si(220) crystal has the advantage of a narrower rocking curve when compared with Si(111), which results in a better collimation (Diat *et al.*, 1997 ▸). Each Si(220) crystal, instead of being part of a channel-cut crystal, is independently precision-mounted on a three-point kinematic mount attached to a rotation stage and controlled by means of an over-constrained weak-link mechanism with picomotor and piezoelectric transducers serving as actuators (Shu *et al.*, 2001 ▸).

We probed the harmonic transmission of the harmonic-rejection crystals using an energy-sensitive Vortex X-ray detector (Hitachi, Chatsworth, CA, USA).^1^ In our experiment, we chose the X-ray energies of 21 keV and 63 keV for the fundamental and harmonic radiation, respectively. We focused on the intensity of the harmonic radiation, and measured the 63 keV intensity with and without the harmonic-rejection crystals in the beam. These results are tabulated in Table 2 ▸.

With the harmonic-rejection crystals out of the beam, the Vortex detector measures the intensity of incoming third-harmonic X-rays from the monochromator as illustrated by the red beam in Fig. 1 ▸. To avoid saturating the detector, we set the beam size at 0.05 mm × 0.10 mm, and used all of the Ti and Al filters available for everyday operation at the beamline as well as two layers of lead tape to attenuate the X-ray intensity. A 5 s exposure produced a detector readout of 1.25 × 10^4^.

With the harmonic-rejection crystals in the beam, the Vortex detector measures the incoming intensity of the harmonic radiation as illustrated by the green beam in Fig. 1 ▸. For this measurement, we used a beam size of 1.00 mm × 1.00 mm and the same amount of beam attenuation. With this beam, a 5 s exposure produced a detector readout of 6. The Vortex detector is a photon-counting device, and its background level is effectively zero. Assuming uniform illumination, a normalization of detector readouts using the beam size yields that the harmonic-rejection crystals have a transmission of 6/(1.00 mm × 1.00 mm)/[1.25 × 10^4^/(0.05 mm × 0.10 mm)] = 2.4 × 10^−6^. Allowing for measurement uncertainties, including those for the Vortex detector counts which exhibit Poisson statistics, this is in reasonable agreement with the theoretical transmission of 3.9 × 10^−6^. It should also be considered that any discrepancy between the measured and predicted values can also arise from the assumptions made concerning the spatial and angular uniformity of the incident X-ray beam, any slight imperfections in the crystal optics (the theoretical calculations assume perfect crystals), as well as imperfect alignment between the two crystals along their tilt directions (Allen *et al.*, 2017 ▸; Ilavsky *et al.*, 2009 ▸).

Although the experiment using an energy-dispersive detector clearly demonstrates that our scheme is highly effective in removing the harmonic radiation, it does not provide information regarding the intensity ratio of fundamental and harmonic radiations in the incoming beam (red beam in Fig. 1 ▸). We performed a second experiment using a photodiode detector and two sets of X-ray filters. By solving a simple set of linear equations, we estimated that in the incoming beam the flux ratio between 63 keV and 21 keV X-rays is 7.3 × 10^−5^ (details about this calculation can be found in the supporting information). Considering the theoretical transmission for the 21 keV X-rays after four reflections from Si(220) crystals is approximately 0.65, we arrived at an estimated flux ratio between 63 keV and 21 keV X-rays in the outgoing beam (green beam in Fig. 1 ▸) of the harmonic-rejection crystal pair to be 2.7 × 10^−10^ using our setup. Importantly, we emphasize that using an X-ray camera we routinely observe positional offset between the fundamental and harmonic radiations emerging from the monochromator. The vertical positional offset observed (typically ∼0.5 mm at the harmonic-rejection crystals) is both a manifestation of the angular dispersion between the fundamental and harmonic radiations, indicated in Fig. 2 ▸(*a*), and is within expectations, given the distance between the monochromator and the harmonic-rejection crystals. This observation provides yet another piece of evidence supporting our proposed harmonic-rejection mechanism.

## 4. Coherence preservation   

For the next-generation synchrotron X-ray sources, one significant breakthrough will be the dramatic increase in beam coherence. As discussed in the *Introduction* [Sec sec1], wavefront distortion introduced by optical elements in the beam path adversely affects coherent X-ray applications such as coherent diffraction imaging and X-ray photon correlation spectroscopy (XPCS). These techniques place a particularly high requirement on the finish of conventional harmonic-rejection mirrors. At the same time, double-crystal optics are known not only to remove unwanted parasitic scattering but also to preserve the coherent wave propagation (Xiao *et al.*, 2006 ▸). Following this concept, we performed XPCS experiments to qualitatively estimate the effect that harmonic-rejection crystals have on the beam coherence.

The setup for these USAXS-based XPCS experiments has been described previously (Zhang *et al.*, 2011 ▸). The sample used for this study was a monodispersed SiO_2_ colloidal suspension, prepared following an established protocol (Zhang *et al.*, 2017 ▸). The diameter of the SiO_2_ particles was approximately 500 nm. The equilibrium dynamics of this colloidal suspension can be characterized using the normalized intensity autocorrelation function *g*_2_(*t*), defined as following its ensemble average:
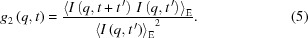
Here, *I*(*q*, *t*) is the time (*t*) dependent detector intensity normalized by ion-chamber readout acquired at a fixed *q.* When the sample contains a large number of independent scatterers undergoing equilibrium thermal motion, following the central limit theorem, the temporal fluctuations of the coherent scattering intensity obey Gaussian statistics and *g*_2_(*q,t*) can be related to the intermediate scattering function of the sample following the Siegert relation (Goodman, 1985 ▸):
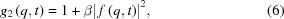
where 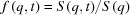 is the normalized intermediate scattering function with *S*(*q*) and *S*(*q,t*) being the static and time-dependent dynamic structure factors at time *t*, respectively. An important conclusion can be drawn from equation (6), which is that as *t*′ → 0, *g*_2_(*q,t*) → 1 + *β*. The parameter *β* is known as the optical contrast and accounts for the smearing that is introduced by scattering from a volume larger than one coherence volume. In other words, *β* provides a quantitative measure that allows the beam coherence to be estimated and compared.

To interrogate the effectiveness that the harmonic-rejection crystals have on preserving the beam coherence, we performed our experiments at 10.5 keV, a previously identified optimal energy for XPCS work at the USAXS beamline (Zhang *et al.*, 2011 ▸). Based on the beam characteristics, we calculated that at 10.5 keV the horizontal and vertical coherence lengths at the beamline are 3.5 µm and 35 µm, respectively.

To test the effect of harmonic-rejection crystal optics on the measured coherence, we used a set of high-resolution JJ slits (JJ slit, Rosskilde, Denmark) as the secondary coherence source, adjusted the vertical and horizontal slit sizes systematically, and characterized the corresponding optical contrasts. The results are shown in Table 3 ▸, from which we conclude that, with the harmonic-rejection optics in the beam at a fixed vertical slit size, increasing horizontal slit size leads to a significant decrease in the optical contrast, owing to the very small value of the horizontal coherence length. In contrast, at a fixed horizontal slit size, the optical contrast is not sensitive to the changes in the vertical slit size until its value greatly exceeds the vertical coherence length. In other words, the beam coherence follows the expected pattern, indicating that the X-ray coherence is preserved, at least qualitatively.

## 5. Conclusions   

In this paper, we have described a simple harmonic-rejection scheme that exploits a mismatch in the crystal Bragg diffractions between those in the monochromator crystals and those in the Bonse–Hart-type harmonic-rejection crystal pair. Our scheme makes use of two reflections by the Si(111) monochromator crystals and four reflections by the Si(220) harmonic-rejection crystals. Dynamical diffraction theory dictates that, for a given crystal, the Darwin curves of fundamental and harmonic radiations have different angular offsets from the Bragg angle θ_B_. Furthermore, their relative offset is different for different crystal reflections. Meanwhile, multiple reflections by single-crystal optics substantially reduce the reflectivity in the tails of the reflectivity curves. By maximizing the transmission of the fundamental radiation, our calculation shows that the allowed transmission of the harmonic radiation (*n* = 3) by the harmonic-rejection crystals is on the order of 10^−6^. Considering an experimentally measured 10^−5^ flux ratio between harmonic radiation and fundamental radiation incident on the harmonic-rejection crystals, our scheme shows that the flux ratio of the harmonic radiation to the fundamental radiation exiting the harmonic-rejection crystals is on the order of 10^−10^. This level of harmonic-rejection is expected to be adequate for the requirements of most, if not all, synchrotron experiments. Minor changes to the monochromator design, making use of four instead of two reflections, would provide an additional five orders of magnitude harmonic rejection.

Using the two-reflection monochromator setup, we performed a qualitative evaluation of the spatial coherence of the X-ray beam while adjusting the beam size systematically. We found that the beam coherence follows the expected behavior, strongly indicating that the coherence of the beam is preserved. This setup is also highly stable. In practice, the X-ray beam after the harmonic-rejection crystals can remain stable for hours without the need of retuning.

We have successfully used this scheme at two synchrotron beamlines at the Advanced Photon Source (sector 9-ID and previously sector 15-ID) to perform small-angle X-ray scattering (SAXS), USAXS, XPCS and imaging experiments. Although both beamlines are equipped with harmonic-rejection mirrors, the mirrors are no longer part of the beamline optics for USAXS operations. These different techniques have very different requirements. Both USAXS and SAXS seek a high dynamic range (over ten decades in scattering intensity in the case of USAXS). Because of this, harmonic contamination is a serious concern (Long *et al.*, 1991 ▸). Using this setup, we performed SAXS using a Pilatus 100K detector and performed USAXS using a high-dynamic-range photodiode detector with an additional pair of Bonse–Hart optics as analyzing crystals. In both types of scattering experiments, no contamination has been detected. We also found that this setup preserves the coherence of the beam for XPCS experiments and removes undesired imaging footprints and distortions caused by the mirror stripes, thus serving to improve data quality and integrity.

When used in place of a harmonic-rejection mirror, this scheme bypasses the increasingly stringent requirements placed on the surface of X-ray mirrors to ensure coherence preservation for applications associated with highly coherent X-rays produced by diffraction-limited storage rings and X-ray free-electron lasers. With an Si(111) monochromator already a required component of many beamlines, the cost for implementing this scheme resides only in the harmonic-rejection crystal pair and its assembly. With the added benefits of coherence preservation and small footprint, we envisage that this highly effective scheme could serve the harmonic-rejection needs of the broad synchrotron community in the era of diffraction-limited storage rings and beyond.

## Supplementary Material

Supporting Information. DOI: 10.1107/S1600577518009645/ve5086sup1.pdf

## Figures and Tables

**Table 1:** Parameters used to calculate the Darwin curve for Si(111) and Si(220) crystals at 21 keV, and Si(333) and Si(660) crystals at 63 keV

	Wavelength (Å)	*d* spacing (Å)	Bragg angle, *θ*_B_ (°)	*f*(0)	*f*(*Q*)	*f′*	*f′′*
Si(111) 21 keV	0.5904	3.1355	5.4023	14	10.540	0.04768	−0.04825
Si(220) 21 keV	0.5904	1.9201	8.8439	14	8.711	0.04768	−0.04825
Si(333) 63 keV	0.1968	1.0452	5.4023	14	6.440	−0.01421	−0.00459
Si(660) 63 keV	0.1968	0.6400	8.8439	14	3.865	−0.01421	−0.00459

**Table 2:** Experimental parameters for measuring harmonic transmission of the harmonic-rejection crystal pair using a Vortex energy-sensitive detector. Uncertainties in the Vortex detector counts follow standard Poisson statistics behavior

	Beam size(mm × mm)	Attenuation	Vortex detector counts of 63 keV X-rays (5 s counting time)
Without harmonic-rejection crystals	0.05 × 0.10	3.75 mm Ti + 3.75 mm Al + 2 layers of lead tape	1.25 × 10^4^
With harmonic-rejection crystals	1.00 × 1.00	3.75 mm Ti + 3.75 mm Al + 2 layers of lead tape	6

**Table 3:** Optical contrast as a function of the horizontal and vertical dimensions of the coherence defining slits

Vertical slit size (µm)	Horizontal slit size (µm)	Optical contrast
15	15	0.044 (2)
15	30	0.031 (2)
15	50	0.018 (1)
15	100	0.0091 (5)
30	15	0.036 (2)
50	15	0.035 (2)
100	15	0.012 (1)
30	30	0.023 (1)
50	50	0.0094 (5)
100	100	0.0067 (3)

## Footnotes

1 Certain commercial equipment, instruments or materials (or suppliers or software) are identified in this paper to foster understanding. Such identification does not imply recommendation or endorsement by the National Institute of Standards and Technology, nor does it imply that the materials or equipment identified are necessarily the best available for the purpose.
