# Supplementary material for: High-efficiency coherence-preserving harmonic rejection with crystal optics
Source: J Synchrotron Radiat. 2018 Aug 14;25(Pt 5):1354–61. doi: 10.1107/S1600577518009645 (PMC6242334; doi:10.1107/S1600577518009645)
Supplement: Supplementary file 1 [file s-25-01354-sup1.pdf]

JOURNAL OF SYNCHROTRON RADIATION

Volume 25 (2018)

Supporting information for article:

High-efficiency Coherence-Preserving Harmonic Rejection with Crystal Optics

Fan Zhang, Andrew Allen, Lyle Levine, Gabrielle Long, Ivan Kuzmenko and Jan Ilavsky

This Supporting Information documents the method we used to measure the flux ratio of the harmonic radiation (n = 3) to the fundamental radiation prior to their entrance to the harmonic-rejection crystal pair. For this analysis, we used Al and Ti X-ray filters. Each set has 4 filters permitting thickness increments in 0.25 mm steps, i.e., either an Al or Ti total thickness of 0.25 mm, 0.50 mm, 0.75 mm, or 1.00 mm. We used a photodiode detector (First Sensor AG*, Berlin, Germany) to detect the transmitted X-ray intensity. This photodiode detector is known to deliver a linear detection response in electrical current to X-ray intensity over a range exceeding 10 orders of magnitude. We placed the monochromator crystals in the beam (after the undulator) to analyze the photon flux of both the fundamental and harmonic radiations after the monochromator. The beamline does not have the capability to handle the white-beam X-rays, hence we cannot quantify the flux of the fundamental and harmonic radiation before the monochromator.

We set the X-ray energy of the fundamental radiation at 21 keV. Hence, the main harmonic contamination concern after the Si (111) monochromator is the presence of the harmonic radiation (n = 3) with an X-ray energy of 63 keV. For a given combination of Al and Ti filters, the X-ray transmissions for 21 keV and 63 keV are different. Thus, we have

$$\begin{aligned} \alpha_1 I_{21keV} + \beta_1 I_{63keV} &= C_1 \\ \alpha_2 I_{21keV} + \beta_2 I_{63keV} &= C_2 \end{aligned} \quad , \qquad (4)$$

where α_1 and β_1 are the X-ray transmission coefficients for 21 keV and 63 keV X-rays for the first filter combination, and α_2 and β_2 at another. C_1 and C_2 are the corresponding detector readouts. Using this pair of linear equations, with α and β calculated from the known filter thickness and X-ray energy, the X-ray intensities of the transmitted fundamental and harmonic radiations can be calculated.

The experimental results are listed in Table S1. We chose the sets of filters to ensure that, even after the monochromator and harmonic rejection crystals, the normalized photodiode readout (photodiode readout normalized by the amplifier gain) is two orders of magnitude above the background level of the photodiode detector ($\approx 2.58 \times 10^{-9}$). At each filter setting, we used a 1 mm × 1 mm beam and counted the photodiode readouts for 5 s. With an incident photon flux at $\approx 10^{13}$ mm^{-2} s^{-1}, this set of acquisition parameters ensures good sampling statistics. We also note that the photodiode has different responses to X-rays of different energies. Following the specification provided by the manufacturer, we estimated that

* **Certain commercial equipment, instruments, or materials (or suppliers, or software, ...) are identified in this paper to foster understanding. Such identification does not imply recommendation or endorsement by the National Institute of Standards and Technology, nor does it imply that the materials or equipment identified are necessarily the best available for the purpose.**

at 21 keV and 63 keV, the X-ray absorption efficiencies for the detector are approximately 29 % and 4 %, respectively.

As summarized in Table S1, we found that, after the monochromator, the flux ratio of 63 keV X-rays to 21 keV X-rays is (0.057/4 %)/(5.70 × 10^3/29 %) = 7.25 × 10^{-5}.

Al filter (mm)	Ti filter (mm)	21 keV Transmission	63 keV Transmission	Photodiode Readout	21 keV Intensity (a.u.)	63 keV Intensity (a.u.)
3.75	0	5.03 × 10^{-2}	7.54 × 10^{-1}	288.2	5.70 × 10^3	0.057
3.75	3.00	3.25 × 10^{-10}	2.96 × 10^{-1}	0.170		

Table S1 Table S1: Photodiode readout and the calculated intensities of the fundamental radiation and harmonic radiation (n = 3) after the Si (111) monochromator.
